# Larger Body Size at Metamorphosis Enhances Survival, Growth and Performance of Young Cane Toads (*Rhinella marina*)

**DOI:** 10.1371/journal.pone.0070121

**Published:** 2013-07-29

**Authors:** Elisa Cabrera-Guzmán, Michael R. Crossland, Gregory P. Brown, Richard Shine

**Affiliations:** School of Biological Sciences A08, University of Sydney, Sydney, New South Wales, Australia; Monash University, Australia

## Abstract

Body size at metamorphosis is a key trait in species (such as many anurans) with biphasic life-histories. Experimental studies have shown that metamorph size is highly plastic, depending upon larval density and environmental conditions (e.g. temperature, food supply, water quality, chemical cues from conspecifics, predators and competitors). To test the hypothesis that this developmental plasticity is adaptive, or to determine if inducing plasticity can be used to control an invasive species, we need to know whether or not a metamorphosing anuran’s body size influences its subsequent viability. For logistical reasons, there are few data on this topic under field conditions. We studied cane toads (*Rhinella marina*) within their invasive Australian range. Metamorph body size is highly plastic in this species, and our laboratory studies showed that larger metamorphs had better locomotor performance (both on land and in the water), and were more adept at catching and consuming prey. In mark-recapture trials in outdoor enclosures, larger body size enhanced metamorph survival and growth rate under some seasonal conditions. Larger metamorphs maintained their size advantage over smaller siblings for at least a month. Our data support the critical but rarely-tested assumption that all else being equal, larger body size at metamorphosis is likely to enhance an individual’s long term viability. Thus, manipulations to reduce body size at metamorphosis in cane toads may help to reduce the ecological impact of this invasive species.

## Introduction

An individual’s body size and mass can affect its viability in many ways; for example, a smaller body often may increase an individual’s vulnerability to mortality sources such as desiccation, predation, competition, starvation or infection [1], [2], [3]. Body size also can influence an animal’s trophic ecology, especially in gape-limited predators [4], [5], [6]. Thus, understanding the consequences of interspecific and intraspecific variation in body size is a key theme of research on life-history evolution [7], [8]. Because an individual’s body size changes considerably during ontogeny, selection can operate not only on size at birth or hatching, but also on size at maturation or on mean adult body size [9], [10], [11]. For species with multiphasic life-histories, body sizes at key developmental stages may be critical determinants of future survival and reproductive success [12], [13]. Because body size can be strongly influenced by ambient conditions (e.g. food supply or temperature), there is abundant opportunity for the evolution of phenotypically plastic norms of reaction that adjust stage-specific growth rates and body sizes to local optima [14], [15], [16], [17].

Some of the best examples of developmental plasticity in body size come from studies on anuran amphibians with aquatic larval stages. The size at which a tadpole metamorphoses not only can trade-off against the duration of the larval period [18], [19], [20], but also can be affected by many attributes of the environment. For example, body size at metamorphosis can be modified by spatially and temporally varying factors such as nutrition [21], [22], larval density [23], [24], temperature [22], [25], falling water levels [26], chemical cues from conspecifics, competitors or predators [24], [27], [28], [29], [30], and pollutants [31]. An extensive literature interprets such shifts as adaptive plasticity, under the hypothesis that natural selection adjusts the timing of metamorphosis in ways that maximize individual fitness [32], [33], [34]. Although the logic is compelling, there are powerful practical impediments to empirically testing this proposition. Measuring lifetime reproductive success requires following individuals throughout their lives, a formidable challenge in taxa with high fecundity and small body sizes at metamorphosis. Even following metamorphs through their early life, to evaluate the degree to which larger size at metamorphosis influences subsequent viability (e.g. survival and growth) is difficult for many species. As a result, the putative advantages of larger body size at metamorphosis remain an assumption for most anuran species, with little empirical support. Notable exceptions include studies on *Pseudacris triseriata* by Smith [35], *Rana sylvatica* by Berven [36], *Bufo terrestris* by Beck and Congdon [37], and *Rana lessonae* and *R. esculenta* by Altwegg and Reyer [12].

The fitness consequences of within-cohort variation in metamorph size are of interest from applied perspectives also. Many anuran species have been widely translocated by humans, and some have become invasive within their new ranges, with substantial ecological impacts on native taxa [38]. As a result, considerable effort has been devoted to developing ways to reduce the abundance of those taxa in their invaded ranges [39], [40], [41]. One possible approach involves targeting the larval stage. Laboratory and field experiments suggest that it may be possible to manipulate (reduce) body size at metamorphosis. This could be done by increasing larval competition with conspecifics (through concentrating oviposition in a smaller number of sites, thus increasing larval density: [42]), or heterospecifics (by encouraging other taxa of anurans to oviposit in the same ponds as used by the target species: [43], [44]. Alternatively, we could use pheromonal cues (from conspecifics, heterospecific anurans, or predators) to induce reaction norms that result in reduced size at metamorphosis [45], [46]. Such manipulations rely upon the assumption that reducing mean body size at metamorphosis will reduce subsequent recruitment of that cohort. To test the validity of that assumption, we need field-based studies on the consequences of metamorphic body size for subsequent rates of survival and growth of the young anurans.

Although intuition suggests that larger body size at metamorphosis should be beneficial, that assumption may not always hold true and it has been rarely documented with field-based data, e.g. [12], [35], [36]. In some anuran species, juvenile frogs can compensate for small metamorph size with enhanced rates of catch-up growth during post-metamorphic life [47], [48]. Thus, the influence of body size at metamorphosis on post-metamorphic survival and growth may differ among species, among local environments within a species, or through time within a single population [37].

In the current study, we build upon extensive research on invasive cane toads (*Rhinella marina*) in the Australian wet-dry tropics. Toad invasion has had major ecological impacts on native predators, stimulating a search for novel methods of toad control [49]. Both in the laboratory and the field, experiments have shown that body size at metamorphosis in cane toads can be reduced by exposure to alarm cues from injured conspecifics [45], [50] and by the presence of tadpoles of native frog species [43]. In other work, smaller body size in immediately post-metamorphic toads has been shown to increase the individual’s vulnerability to desiccation [51], [52], to mortality caused by lungworm infection [53], and to predation by cannibalistic conspecifics [54] and predatory ants [55]. In order to evaluate the plausibility of reducing toad recruitment by reducing body sizes at metamorphosis, we conducted experimental trials to measure the effects of body size (mass) at metamorphosis on growth rates, survival, and performance of metamorph and juvenile cane toads. The experimental trials were conducted in outdoor enclosures to test growth and survival of individuals under natural conditions, and in the laboratory to test performance under controlled conditions.

## Materials and Methods

### Ethics Statement

The study was performed at the Tropical Ecology Research Facility (University of Sydney) located at Middle Point, Northern Territory, Australia (see below for further details). The Facility does not belong to a National Park or other protected area of land. Permission for research on amphibian species at this location was granted by the Parks and Wildlife Commission of the Northern Territory (permit numbers 36597 and 40463) and the University of Sydney Animal Ethics Committee (Protocol L04/6-2010/3/5333). The study was conducted with an invasive species (the cane toad: *Rhinella marina*) and with native frog species that are not endangered. Two egg clutches of cane toads (*R. marina*) and egg clutches of the native frogs were collected by hand from natural ponds. Another six cane toad egg clutches were obtained by injecting hand-collected adult toads (six male, six female) with 0.25 mg/mL of leuprorelin acetate. No animals were killed. All procedures were approved by the University of Sydney Animal Ethics Committee (Protocol L04/6-2010/3/5333).

### Study Species and Site

The cane toad (*Rhinella marina*, Linnaeus 1758), native to a wide area of the Americas (from the southern USA to Brazil, South America), was introduced to north-eastern Australia in 1935 as a biological control agent of insect pests of sugar cane [56], [57]. As cane toads have spread throughout tropical and subtropical Australia, they have killed many native predators that attempt to prey on these toxic anurans [49], [58]. The cane toad life cycle includes aquatic eggs and larvae and terrestrial metamorphs, juvenile and adults. All of these stages contain toxic bufadienolides, although toxin type and quantity vary through toad ontogeny [59].

The Adelaide River floodplain, 60 km east of Darwin in the wet-dry tropics of Northern Territory (12°42′43″S, 131°18′52″E) was first colonized by cane toads in 2005 [56]. In this area, air temperatures are high and stable (maximum monthly temperatures >30°C year-round), whereas minimum (overnight) temperatures are lower mid-year. Monsoonal rainfall is highly seasonal with more than 75% of the annual rainfall coming in less than four months, usually starting in December and peaking in the period from January to March [60], [61]. This seasonality causes major changes to the landscape, with the formation of temporary water bodies and a rapid increase of vegetation growth and animal activity during the wet season (M. R. Crossland and E. Cabrera-Guzmán unpublished data). Cane toads breed in temporary and permanent ponds across this area, spawning most frequently at the end of the wet-season, as pond levels begin to fall; but they also can breed during the dry season [42], (M. R. Crossland and E. Cabrera-Guzmán unpublished data). Toad metamorphs (the first terrestrial phase, immediately after transformation from the tadpole) are mainly diurnal [62] and remain near the edge of natal ponds during the dry season [63]. They disperse through the wider landscape during the wet season [51], [63], [64] but often remain close to the pond from which they emerged [52]. Juvenile toads become increasingly nocturnal. Post-metamorphic toads feed mostly on small insects such as ants and beetles [64], [65], [66].

### Locomotor Performance and Feeding Ability

We reared toad tadpoles at different densities in two trials to produce toad metamorphs of different body sizes. Tadpoles were reared in plastic bins (60×40×40 cm), each filled with 65 L of bore water and a substrate of dirt collected from natural temporary ponds. We initially added 1–1.5 g of crushed algae wafers (Hikari tropical algae wafers, Kyorin Food Industries Ltd, Chikugo, Japan: crude protein 33%, crude fat 4%, crude fibre 3%, moisture 10%, crude ash 17%, phosphorus 0.8%) to each bin as a food source for tadpoles. For the first trial (November 2010), we obtained two naturally-spawned clutches from local waterbodies: one of *R. marina* and one of the native ornate burrowing frog (*Opisthodon ornatus* Gray, 1842). We reared cane toad tadpoles at two densities: (1) 10 *R. marina* tadpoles per container, (2) 10 *R. marina* tadpoles plus 10 *O. ornatus* tadpoles per container. For the second trial (December 2011), we obtained one cane toad clutch by injecting local field-collected adults (one male, one female) with 0.25 mg/mL of leuprorelin acetate (Lucrin, Abbott Australia), to induce amplexus and spawning. The resulting tadpoles were allocated to two density treatments: 10 or 20 *R. marina* per tub. Each rearing trial was a randomized block design with four replicates per treatment.

We checked the containers daily and collected individuals with emerged forelimbs over a two-week period after the first metamorph had emerged. We tested a total of 60 metamorphs: 36 from the first larval trial and 24 from the second one. These animals were kept in 1 L rectangular plastic containers with a small amount of water. On the day metamorphosis was complete (defined as total absorption of the tail), we conducted performance trials between 1000 and 1400 h (mean temperature and humidity: 29.8°C, 60.6%) in a shaded building at the University of Sydney Tropical Ecology Research Facility, Middle Point (12°34′44″S, 131°18′51″E). For each individual, we tested jumping performance on the concrete floor by touching the urostyle of the metamorph with a wooden stick (50 cm length) to induce it to jump. We recorded the distance of each of the first three jumps (measured with a ruler from the damp “prints” produced by the metamorph). After 5 minutes rest in its container, we tested the individual’s swimming performance in a water-filled trough (112 cm long, 12 cm wide, 10 cm high, water 5 cm deep). We recorded the total distance swum by the metamorph in 2 minutes. After the swimming trial, we waited another 5 minutes before evaluating feeding performance. We placed the metamorph plus 30 live termites in a plastic container (34×22×10 cm) on a layer of wet dirt, and recorded the number of termites eaten during the next 5 minutes. We measured the snout-urostyle length (SUL) and weight of each metamorph with electronic digital calipers and an electronic balance (Scout Pro 200 g, OHAUS, Pine Brook, NJ) at the completion of the performance and feeding trials. We did not measure metamorphs prior to these trials because pilot studies showed that handling can induce abnormal behaviour. These pilot studies also showed that metamorphs that ate up to six termites increased in mass by <0.05%; therefore, the prey consumed during a trial (metamorphs never consumed more than six termites per trial) had a negligible effect on their mass.

### Rates of Growth and Survival

We performed three mark-recapture trials to assess the consequences of body size at metamorphosis on growth and survival. Metamorphs were obtained from laboratory experiments on larval competition. The experiments were performed in 65 L or 15 L plastic bins, with natural substrata and crushed vegetable wafers as larval food. One experiment was conducted in May 2009 (“Experiment 1”) and one in January 2010 (“Experiment 3”). We used a total of four and eight treatments respectively, with either 10 larval *R. marina* per container, or 20 *R. marina* per container, or 10 *R. marina* plus 10 tadpoles of a native species. We used eight species of native tadpoles in these trials (see [43] for a detailed description of experiments and results; n = 4 cane toad clutches). Another experiment, performed in September 2009 (“Experiment 2”), comprised eight treatments with lower numbers of tadpoles per container (n = 5 *R. marina*, 10 *R. marina*, or 5 *R. marina* plus 1 tadpole of a native species; n = 2 cane toad clutches). Most of the smaller metamorphs were from treatments where toad tadpoles were exposed to native tadpoles or to high densities of conspecific tadpoles, whereas the larger metamorphs came from treatments with less larval competition. The metamorphs used in the growth and survival trials emerged from rearing tubs over an eight-day period. As metamorphs emerged, they were collected and kept in 1 L rectangular plastic containers with a little water and fed with termites three times weekly for one or two weeks depending on their dates of emergence.

The three mark-recapture trials were performed from June to August 2009 (Trial 1), October to November 2009 (Trial 2), and February to August 2010 (Trial 3). In each trial, metamorphs were released into three large (15×4 m) adjacent open-topped outdoor enclosures with similar conditions and resources to those present in and around nearby natural waterbodies. The walls of these enclosures were formed by metal sheets (0.8 m high) and were located within the grounds of the Tropical Ecology Research Facility. Each enclosure housed a pond (2.5×1.8×0.6 m) that contained, and was surrounded by, natural vegetation. The ponds and enclosures had been constructed 4 years prior to our trials, and had already been colonized by native anurans (*Crinia* sp., *Limnodynastes convexiusculus*, *Litoria australis, L. bicolor, L. dahlii*, *L. nasuta*, *L. rothii, L. rubella*), as well as by other potential prey, competitors, and predators (e.g. ants, beetles, bugs, spiders, centipedes, worms, snails, snakes and birds). Piles of dry grass 10 cm from the water of each pond provided pondside refuges for the metamorphs.

At the beginning of each trial we recorded body length (SUL) and mass of each metamorph. Body size was recorded with electronic digital calipers and mass was measured with an electronic balance (Scout Pro 200 g, OHAUS, Pine Brook, NJ). The range of body sizes was 7.4–13.5 mm SUL, and 0.03–0.25 g body mass. For Trial 1, we individually marked 63 toads by toe clipping (up to 3 toes carefully clipped with ophthalmological scissors) and these individuals were released around the ponds into the field enclosures the following day. Each of the enclosures was allocated one-third of the animals (21 metamorphs per enclosure for Trial 1). We followed the same methodology for Trials 2 and 3, with 94 and 148 metamorph toads respectively. Every 2 weeks we exhaustively searched for the metamorph and juvenile toads, during both day and night and collected them to record individual identity, SUL, and mass before returning them to their enclosures the following day. The duration of each of the three trials differed, dependent upon mortality rates. The number of recaptured metamorphs declined rapidly during the first two trials, but not in Trial 3; thus the duration in the last trial was greater (see below; Trial 1 = 65 days, Trial 2 = 40 days, Trial 3 = 185 days). Records of air temperature and rainfall during the mark-recapture period were obtained from an automated weather station located <5 km from the enclosures.

### Statistical Analyses

In a preliminary covariance analysis with metamorph mass as a covariate and trial # as the factor, we found no significant effects of trial # on swimming or feeding performance (*P*>0.05), and a marginally significant effect of trial # for jumping performance (but with similar trends in both sets of trials). Thus we combined the results obtained in both trials and used linear regression to compare the performance of metamorphs of different sizes, with metamorph mass as the independent variable and mean distance jumped, total distance swam, and total number of termites consumed as response variables. We compared the average rainfall (ln –transformed) and air temperature among trials data using ANOVA.

For toads recaptured during the outdoor-enclosure trials, we calculated daily growth rates as ([metamorph mass at recapture – initial mass]/# days in enclosure). We used multiple regression to assess the effects of initial mass, climate variables and Trial # on growth rates and included toad ID (identity) as a random effect. We also repeated the analysis using larval treatment group as an independent variable, instead of initial body mass, to determine which variable better explained growth rate variation. Models containing both initial mass and larval treatment groups were over-parameterized or too unbalanced to run. To assess whether the growth advantage conferred by larger initial size was retained over time, for each Trial we carried out an ANCOVA with growth rate as the independent variable and with initial mass as the covariate and recapture period as a factor. For all three trials the first recapture period occurred after 16–20 days, the second after 30–34 days, the third after 40–51 days, and, for Trial 3, a final recapture took place after 185 days. Because some individuals were captured during more than one period, we included ID as a random effect in these ANCOVAs. All analyses were performed using JMP 9 software (SAS Institute, Cary, NC).

Survival was estimated using the Cormack-Jolly-Seber model for mark-recapture experiments with MARK 6.0 software [67], [68], [69]. In addition to standard model forms where survival and recapture parameters were constant or time-dependant we constructed models where both parameters were constrained by the initial mass of each toad. We also repeated the analyses, but instead of constraining the survival and recapture with initial mass, they were constrained by larval treatment groups. We assessed model fit using Akaike’s Information Criterion (AIC) values, with lower values indicating models with greater support [70].

## Results

### Locomotor Performance and Feeding Ability

**Figure 1:**
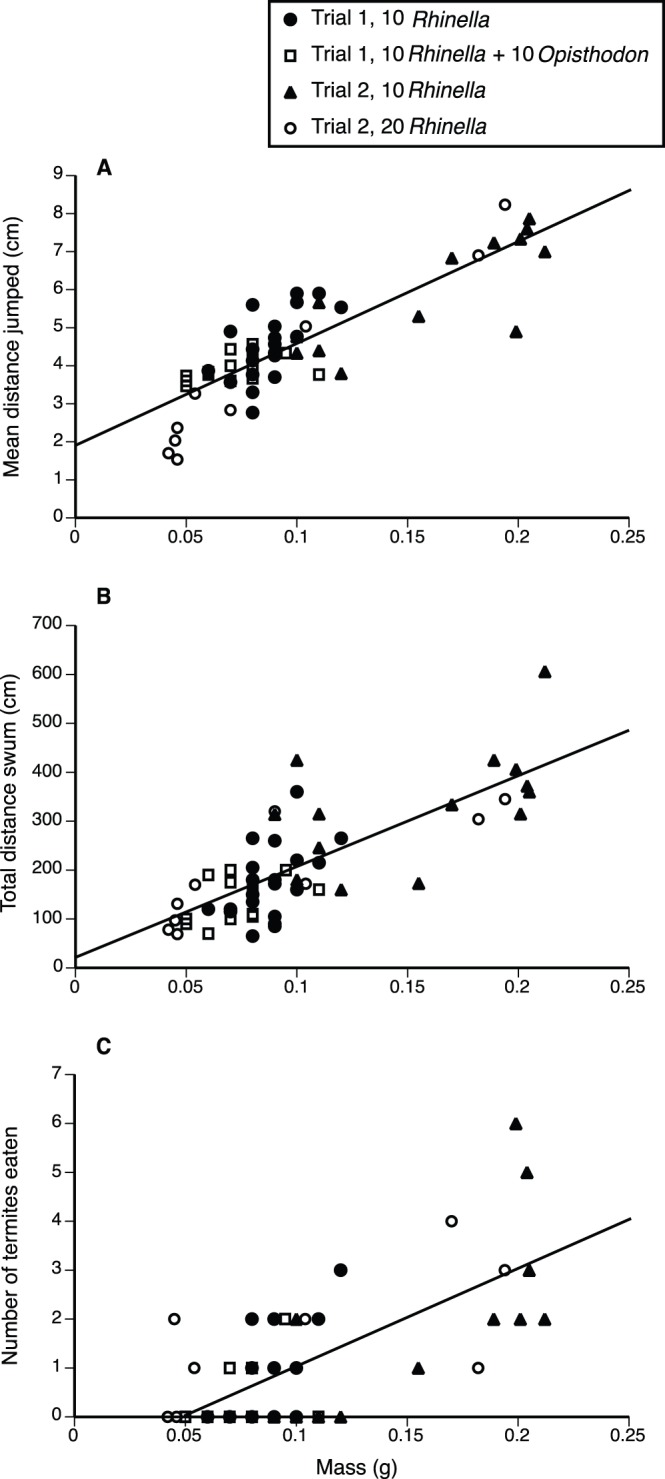
Relationships between the body mass of a metamorph cane toad and its performance. Performance was measured by (A) mean distance of the three first jumps, (B) total distance swum, and (C) total number of termites eaten.

Larger metamorph toads jumped further than did their smaller siblings (*R* ^2^ = 0.737, *F*_1,58_ = 163.33, *P*<0.0001; Figure 1A), swam greater distances (*R* ^2^ = 0.608, *F*_1,58_ = 90.25, *P*<0.0001; Figure 1B) and ate more prey items (*R* ^2^ = 0.467, *F*_1,58_ = 50.88, *P*<0.0001; Figure 1C).

### General Patterns in Growth and Survival

In Trial 1, 63 metamorphs were released into the outdoor enclosures (Day 0), with 30 of them recaptured on Day 15, 17 on Day 30, 12 on Day 51 and 8 on Day 65. Of the 94 metamorphs released in Trial 2, 35 were recaptured on Day 16, 14 on Day 30 and 4 on Day 40. In Trial 3, 148 metamorphs were released, 33 were recaptured on Day 10, 19 on Day 20, 19 on Day 34, 13 on Day 48, 30 on Day 134, and 28 on Day 185. Some of the individuals in this last trial were recaptured as juveniles (from 30–90 mm SUL), based on the classification of [71] and [72].

Average air temperatures differed significantly among the three trials (ANOVA *F*_2,285_ = 68.16, *P*<0.0001). Trial 2 was the warmest (30.1°C) followed by Trial 3 (27.1°C), and Trial 1 was the coolest (24.4°C). Rainfall also varied significantly among trials (ANOVA *F*_2,278_ = 8.26, *P* = 0.0003). Trial 1 was conducted during the dry season and experienced only 0.2 mm of cumulative rainfall. Trial 2 (conducted immediately prior to the wet season) experienced a total of 83.6 mm rainfall while during Trial 3 (conducted on the wet-season), the study site experienced 338.2 mm rainfall.

**Figure 2:**
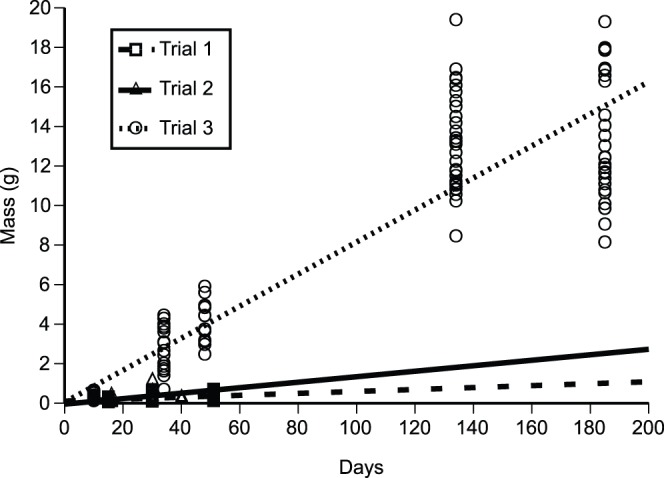
Growth trajectories (changes in mass over time) for metamorph and juvenile cane toads in three trials conducted in outdoor enclosures.

Metamorph and juvenile toads grew more rapidly in Trial 3 than in Trials 1 and 2. At the same ages, toads in Trial 3 were much larger (mean mass at 34 days = 2.6 g, mean mass at 48 days = 4.1 g) than toads in Trial 1 (mean mass at 30 days = 0.23 g, mean mass at 51 days = 0.36 g) and Trial 2 (mean mass at 30 days = 0.47 g, mean mass at 40 days = 0.29 g). In these latter two trials, some young toads in the outdoor enclosures failed to grow during their first month post-release, and a few decreased in mass over this period. At Day 30, toads in Trial 1 had, on average, not gained any mass, toads in Trial 2 were twice their initial mass, and toads in Trial 3 were 20 times heavier than at the time of release (Figure 2).

### Effects of Trial and Initial Mass on Growth Rates

An initial model, testing the effects of Trial #, initial mass, air temperature and rainfall on growth rates revealed a significant interaction between Trial # and air temperature (*F*_2,193_ = 4.21, *P = *0.016). This interaction indicated that the effects of temperature on growth differed among the three trials; therefore we carried out subsequent analyses on each trial separately. In Trial 1, there were no significant effects of initial mass, air temperate or rainfall on growth rate (all *F*_1,35_<1.44, all *P*>0.24). When larval treatment group was substituted into the model in place of initial mass, it was non-significant also (*F*_2,28_ = 1.11, *P* = 0.34).

During Trial 2, growth rate was affected by initial mass (*F*_1,41_ = 27.8, *P*<0.0001) and air temperature (*F*_1,34_ = 37.1, *P*<0.0001) but the effect of rainfall was marginally non-significant (*F*_1,41_ = 3.9, *P* = 0.06). When larval treatment group was substituted into this analysis in place of initial mass, it was significant (*F*_7,23_ = 4.6, *P* = 0.002), but had much less explanatory power than did initial mass.

In Trial 3, growth rate increased with initial mass (*F*_1,35_ = 5.5, *P* = 0.025), temperature (*F*_1,103_ = 20.9, *P*<0.0001) and rainfall (*F*_1,93_ = 25.8, *P*<0.0001). The effect of larval treatment group was non-significant (*F*_5,37_ = 1.7, *P* = 0.16) when it replaced initial mass in the analysis.

**Figure 3:**
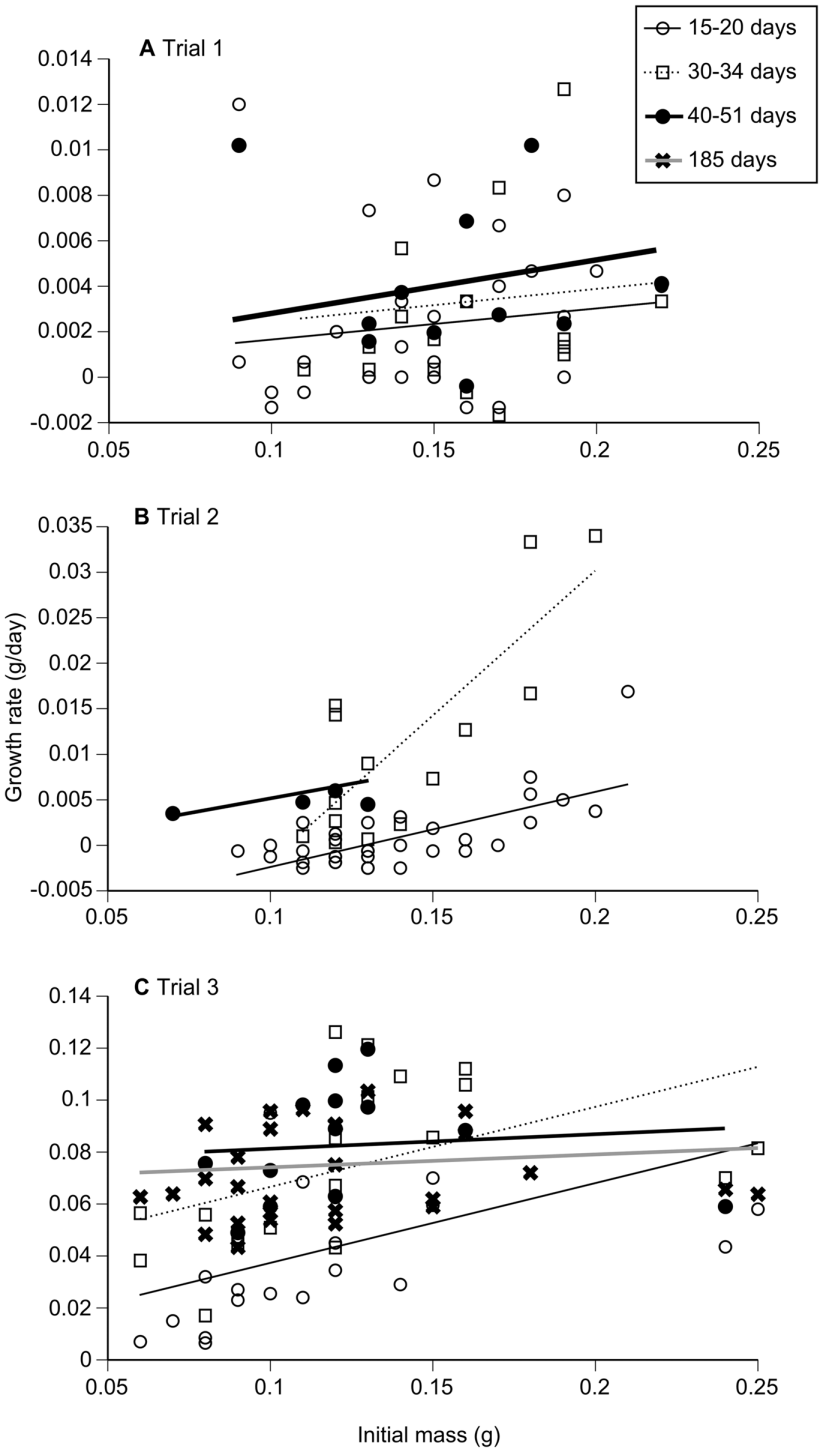
Relationship between initial body mass and growth rate of metamorph and juvenile cane toads during Trial 1 (A), Trial 2 (B), and Trial 3 (C) over different recapture periods.

During Trial 1, growth rates differed among the three recapture periods (*F*_2,25_ = 6.29, *P = *0.006), being lowest during the first period, intermediate during the second, and highest during the third period. However, growth rate was not significantly related to initial mass (*F*_1,32_ = 0.53, *P* = 0.47) nor was there an interaction between initial mass and period (*F*_2,25_ = 0.22, *P* = 0.80) (Figure 3A). During Trial 2 there was a significant interaction between initial mass and period (*F*_2,37_ = 16.81, *P*<0.0001) indicating that the slope of the relationship between initial mass and growth rate varied among the recapture periods. Inspection of the interaction plot (Figure 3B) revealed that the positive effect of initial mass on growth rate was stronger (steeper slope) during the second recapture period. During Trial 3 there was again a significant interaction between initial mass and growth rate (*F*_3,38_ = 4.47, *P* = 0.009). Inspection of the interaction plot indicated that the relationship between growth rate and initial mass was steep during the first two periods, but levelled off during the third and fourth recapture periods (Figure 3C).

### Effects of Initial Mass on Survival Rates

**Figure 4:**
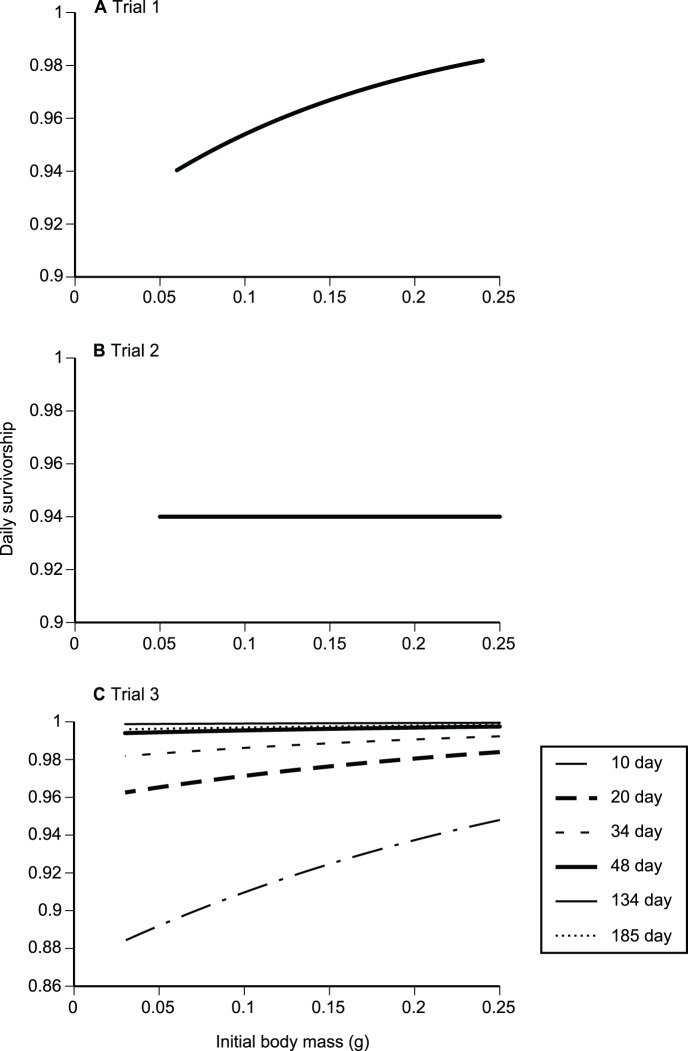
Effects of initial body mass on survival of cane toad metamorphs in outdoor enclosures in (A) Trial 1, (B) Trial 2, and (C) Trial 3. For (C) the lower line indicates the first time period (10 days), the second line indicates the second time period (20 days) and the highest line indicates the last period (sixth recapture period: 185 days).

We fitted data separately for each trial. For Trial 1, the survival probability of a juvenile toad increased with its initial mass, as indicated by the top three models in the mark-recapture analysis (Table 1; Figure 4A). Daily survival probability was 0.94 for the smallest toads, and 0.98 for the largest toads. For Trial 2, survival was best modelled as a constant value of 0.94, unrelated to initial mass (Table 1; Figure 4B). In Trial 3, the best models showed survival increasing with initial mass, and varying through time. The positive relationship between survival and initial size decreased as the trial progressed, and overall survival rate increased (Table 1; Figure 4C).

**Table 1:** Model output (three most parsimonious models for each trial) and parameter estimates for the mark-recapture data from metamorph and juvenile cane toads (*Rhinella marina*) in outdoor enclosures in tropical Australia.

Trial	Model	AICc	Delta AICc	AICc Weights	Model Likelihood	Number of parameters
1	Survival (mass)Recapture (time)	205.7622	0	0.2140	1	4
1	Survival (mass)Recapture (mass+time)	207.1752	1.413	0.1056	0.4934	5
1	Survival (mass)Recapture (constant)	207.3882	1.626	0.0949	0.4435	3
2	Survival (constant)Recapture (mass*time)	192.6065	0	0.4682	1	3
2	Survival (time)Recapture (mass*time)	194.3823	1.7758	0.1926	0.4115	4
2	Survival (mass*time)Recapture (mass*time)	196.2574	3.6509	0.0754	0.1611	6
3	Survival (mass+time)Recapture (mass+time)	540.8827	0	0.5404	1	13
3	Survival (time)Recapture (time)	542.9603	2.0776	0.1912	0.3539	11
3	Survival (time)Recapture (mass+time)	543.4182	2.5355	0.1521	0.2815	13

In parallel analyses using larval treatment as a grouping variable instead of initial mass, the best fitting models in which larval group affected survival had much higher AIC values than the best models constrained by initial mass (Trial 1∶210.6 vs 205.8, Trial 2∶213.4 vs 192.6, Trial 3∶595.7 vs 540.9).

## Discussion

Our data support a commonly-made but rarely-tested assumption: that increased body size at metamorphosis in anurans is associated with traits that definitely (in the case of survival) or plausibly (in the case of growth, locomotor performance and feeding ability) enhance individual fitness. Smaller body size also renders a young cane toad more vulnerable to desiccation [51], [52], predation by carnivorous ants [55], cannibalism [54], and mortality after lungworm infection [53]. Thus, for this species, we can confidently conclude that (a) toad body size at metamorphosis can be reduced by multiple biotic and abiotic characteristics of the larval environment [43], [44], [45], [46], (b) that any reduction in body size at metamorphosis is likely to impose subsequent fitness costs, and (c) the strength of any link between metamorph mass and subsequent survival and growth rates will vary through time and space, at least partly because of variation in the availability of environmental resources.

Our laboratory-based results showing performance advantages to larger body size at metamorphosis are unsurprising: an abundant literature demonstrates that larger size usually (but not always) confers performance advantages in a diverse array of functions in anurans such as metabolic rates and endurance, jumping ability (larger individuals having longer jump length, or showing higher endurance and speed), feeding (higher attack rates on prey, higher prey capture success, and wider prey size range) and escape from predators, e.g. [19], [55], [73], [74], [75], [76], [77]. Thus, larval history (particularly effects on body size caused by high levels of stress by competition and predation) can reduce the chances of survival for post-metamorphic and juvenile anurans [12], [23], [78], [79]. In amphibians, larger body size can enhance fitness not only via natural selection [80], but also via sexual selection in adult males (e.g. time to maturity: [36], [81], [82]), fecundity selection in adult females [83], [84], [85], [86], and size at first reproduction in both, males and females [87]. Bigger generally seems to be better, albeit with exceptions: for example, larger metamorph size in the frog *Limnodynastes convexiusculus* can actually decrease rather than increase survival, because larger maximum gape size increases the ability of a metamorph frog to ingest a (fatally toxic) metamorph cane toad [88].

Our mark-recapture results provide the first robust evidence of survival and growth advantages to larger metamorph size in cane toads under semi-natural conditions. Although we monitored toad viability in outdoor enclosures rather than around natural ponds, the large size of our enclosures, and the diverse native fauna they contained (reflecting easy ingress and egress by most native fauna) facilitate extrapolation to the wild. The abiotic and biotic conditions inside our enclosures resembled those around nearby waterbodies in which cane toads frequently spawn, although natural ponds are likely to be inhabited by more predators including a wider size range of toads, snakes, wading birds and parasites such as lungworms in the genus *Rhabdias*.

Our results highlight the sensitivity of toad growth rates to seasonally varying conditions. During the wet-season (Trial 3) when food and water were abundant, the young toads grew very quickly (mean of 20-fold increase in initial mass over a month). In contrast, growth was negligible over the same period during the dry-season (mean of 0.5-fold increase in mass in Trial 1). Growth rate was positively related to rainfall and air temperature during Trial 3. Moist substrates during the wet-season not only remove hydration stress, but also allow the young toads to forage widely instead of remaining close to the pond edge where the density of conspecifics (and thus, competition for food) is greater [51], [64], [71]. Also, invertebrate prey (mostly insects) are more abundant and more active during the wet-season [51]. The striking differences between toad growth rates in wet-season versus dry-season trials reinforce the importance of seasonally variable precipitation regimes for anuran ecology in the wet-dry tropics (see also [89]) and the important role of rainfall for amphibian post-metamorphic growth and survival [90].

Under dry-season conditions when growth is slow or negligible, there is little opportunity for growth rates to be affected by other factors such as body size at metamorphosis. However, the more rapid growth seen during the wet-season allows abundant opportunity for us to detect other influences on rates of growth. In our field enclosures, low body mass at metamorphosis (as induced by exposure to competitors during the larval stage) continued to affect toad metamorph body size for at least a month post-metamorphosis in Trials 2 and 3. This advantage weakened through time, with no detectable effect of initial body mass on growth rates of larger juvenile toads. The reduction in effect may have several causes. For example, the effects of individually variable phenotypic traits that influence growth rate may become increasingly important relative to initial size. Also, high mortality rates of the smallest animals increasingly remove the lower tail of the body-size distribution, leaving only a subset of individuals that were all relatively large at metamorphosis. Within that relatively restricted size range, initial mass may cease to be a strong predictor of an individual’s subsequent growth trajectory.

Low growth rates under stressful dry-season conditions reduce the overall range of body sizes within a cohort of young toads, whereas favourable conditions result in a much wider size range. For example, at the end of one month of growth, young toads in Trial 1 weighed from 0.12–0.57 g (a range of 0.45 g) and from 0.13–1.22 g (range 1.09 g) in Trial 2, whereas young toads in Trial 3 weighed from 0.66–4.41 g (a range of 3.75 g). Asymmetry in the sizes of conspecifics increases the probability of cannibalism [6]. In pondside aggregations of post-metamorphic cane toads during the dry-season, individuals that grow large enough to ingest the smallest of their siblings switch to a cannibalistic diet [54]. Thus, the high growth rates enabled by favourable climatic conditions will create an additional mortality risk based on body size, strengthening the advantage to larger body size at metamorphosis. In this sense, the dynamics of cannibalism in juvenile cane toads are the opposite of those documented in cannibalistic larvae of the toad *Scaphiopus bombifrons* and salamanders *Ambystoma macrodactylum*, *A. tigrinum nebulosum* and *Hynobius retardatus*, where low food supply and thus hunger (or inducible defences in heterospecific prey) stimulates some individuals to switch their diets towards cannibalism [6], [91], [92], [93], [94]. Facultative cannibalism in early life-history stages of anurans thus may arise as a result of unusually high growth rates (in cane toads) as well as unusually low growth rates (in *Scaphiopus bombifrons* and the salamanders noted above).

The link between growth rates and opportunities for cannibalism (above) is not the only mechanism creating a relationship between rates of survival and growth. A more direct relationship is that low feeding rates reduce both survival and growth. In Trials 1 and 3, we found that the smallest individuals at metamorphosis were unlikely to survive. Our data do not identify the factors causing mortality, but these likely involve interactions between predation, cannibalism, parasitism, desiccation and thermal variation [51], [52], [53], [54], [55], [64], [71]. Predation is likely to have been an important mortality source. We found several dead frogs (*Litoria dahlii*) around the ponds, strongly suggesting that they had ingested toxic metamorphic toads [95]. We also observed predation by large ants (giant snappy ant - *Odontomachus turneri*) on marked toad metamorphs. Under some conditions ants can decimate cane toad populations, with smaller metamorph toads being more vulnerable to predatory ants than are larger conspecifics [55].

Lastly, we consider the implications of our results for the idea that reducing toad size at metamorphosis may help to reduce rates of recruitment of this troublesome invasive species. Size reduction could be achieved by pheromonal means [45], [46], by concentrating toad oviposition in a smaller number of ponds and thus increasing larval densities [42], by increasing parasite uptake [53], or by encouraging native frogs to spawn in the same ponds as the toads, thereby intensifying larval competition [43], [44]. Controlling the metamorph stage can be an effective method to decrease population growth rates in invasive bullfrogs [96]. Encouragingly, our study suggests that toads metamorphosing at smaller sizes are likely to experience reduced survival rates and to remain smaller than usual for at least the first month of their life, a critical period in the cane toad life cycle. Smaller size may render a metamorph toad more easily ingestible by small species of native predators (because of gape-limitation), thus increasing risk to individuals of such species, but the overall ecological impact of cane toads on such taxa is minor [49]. For the native species most at risk from toad invasion (large predators such as quolls, and varanid and scincid lizards), reducing toad size at metamorphosis may confer two advantages. First, smaller toads are less toxic, so that consuming one may teach large predators not to attack larger more deadly toads [97]. Second, reduced size at metamorphosis likely will reduce overall survival rates of toads, thus reducing the numbers of (deadly) adult toads recruiting into the system. Further work is needed to quantify elasticities in toad recruitment, however, because density-dependent processes may weaken any relationship between metamorph abundance and the numbers that survive into the adult stage.
